# DNA methylation age of blood predicts all-cause mortality in later life

**DOI:** 10.1186/s13059-015-0584-6

**Published:** 2015-01-30

**Authors:** Riccardo E Marioni, Sonia Shah, Allan F McRae, Brian H Chen, Elena Colicino, Sarah E Harris, Jude Gibson, Anjali K Henders, Paul Redmond, Simon R Cox, Alison Pattie, Janie Corley, Lee Murphy, Nicholas G Martin, Grant W Montgomery, Andrew P Feinberg, M Daniele Fallin, Michael L Multhaup, Andrew E Jaffe, Roby Joehanes, Joel Schwartz, Allan C Just, Kathryn L Lunetta, Joanne M Murabito, John M Starr, Steve Horvath, Andrea A Baccarelli, Daniel Levy, Peter M Visscher, Naomi R Wray, Ian J Deary

**Affiliations:** Centre for Cognitive Ageing and Cognitive Epidemiology, University of Edinburgh, 7 George Square, Edinburgh, EH8 9JZ UK; Medical Genetics Section, Centre for Genomic and Experimental Medicine, Institute of Genetics and Molecular Medicine, University of Edinburgh, Edinburgh, EH4 2XU UK; Queensland Brain Institute, The University of Queensland, Brisbane, 4072 QLD Australia; University of Queensland Diamantina Institute, Translational Research Institute, The University of Queensland, Brisbane, 4072 QLD Australia; The NHLBI’s Framingham Heart Study, Framingham, MA 01702 USA; Population Sciences Branch, Division of Intramural Research, National Heart, Lung, and Blood Institute, Bethesda, MD 01702 USA; Department of Environmental Health, Harvard T.H. Chan School of Public Health, Boston, MA 02115 USA; Wellcome Trust Clinical Research Facility, University of Edinburgh, Western General Hospital, Crewe Road, Edinburgh, EH4 2XU UK; Queensland Institute of Medical Research Berghofer Medical Research Institute, Brisbane, 4029 QLD Australia; Department of Psychology, University of Edinburgh, Edinburgh, EH8 9JZ UK; Center for Epigenetics, Johns Hopkins University School of Medicine, Baltimore, MD 21205 USA; Departments of Medicine, Molecular Biology/Genetics, Oncology, and Biostatistics, Johns Hopkins University School of Medicine, Baltimore, MD 21287 USA; Department of Mental Health, Johns Hopkins University Bloomberg School of Public Health, Baltimore, MD 21205 USA; Lieber Institute for Brain Development, Baltimore, MD 21205 USA; Harvard Medical School, Boston, MA 02115 USA; Hebrew Senior Life, Boston, MA 02131 USA; Department of Epidemiology, Harvard T.H. Chan School of Public Health, 665 Huntington Ave, Boston, MA 02115 USA; Department of Biostatistics, Boston University School of Public Health, Boston, MA 02118 USA; Section of General Internal Medicine, Department of Medicine, Boston University School of Medicine, Boston, MA 02118 USA; Alzheimer Scotland Dementia Research Centre, University of Edinburgh, Edinburgh, EH8 9JZ UK; Human Genetics, Gonda Research Center, David Geffen School of Medicine, University of California Los Angeles, Los Angeles, CA 90095-7088 USA; Biostatistics, School of Public Health, University of California Los Angeles, Los Angeles, CA 90095 USA

## Abstract

**Background:**

DNA methylation levels change with age. Recent studies have identified biomarkers of chronological age based on DNA methylation levels. It is not yet known whether DNA methylation age captures aspects of biological age.

**Results:**

Here we test whether differences between people’s chronological ages and estimated ages, DNA methylation age, predict all-cause mortality in later life. The difference between DNA methylation age and chronological age (Δ_age_) was calculated in four longitudinal cohorts of older people. Meta-analysis of proportional hazards models from the four cohorts was used to determine the association between Δ_age_ and mortality. A 5-year higher Δ_age_ is associated with a 21% higher mortality risk, adjusting for age and sex. After further adjustments for childhood IQ, education, social class, hypertension, diabetes, cardiovascular disease, and *APOE* e4 status, there is a 16% increased mortality risk for those with a 5-year higher Δ_age_. A pedigree-based heritability analysis of Δ_age_ was conducted in a separate cohort. The heritability of Δ_age_ was 0.43.

**Conclusions:**

DNA methylation-derived measures of accelerated aging are heritable traits that predict mortality independently of health status, lifestyle factors, and known genetic factors.

**Electronic supplementary material:**

The online version of this article (doi:10.1186/s13059-015-0584-6) contains supplementary material, which is available to authorized users.

## Background

DNA sequence variants and epigenetic marks that are associated with changes in gene expression contribute to interindividual variation in complex phenotypes. Epigenetic mechanisms such as DNA methylation, characterized by the addition of a methyl group to a cytosine nucleotide primarily at cytosine-phosphate-guanine (CpG) sites, play essential roles during development, acting through the regulation of gene expression [1]. Unlike genomic variants, such as single nucleotide polymorphisms (SNPs), levels of DNA methylation vary across the life course [2-6]. DNA methylation levels are influenced by lifestyle and environmental factors [7], as well as by genetic variation [8,9].

Age-related changes in DNA methylation are also well documented, and two recent studies used methylation measures from multiple CpG sites across the genome to predict chronological age in humans [10,11]. Hannum *et al.* created an age predictor based on a single cohort in which DNA methylation was measured in whole blood [10]. Horvath developed an age predictor using DNA methylation data from multiple studies (including the Hannum dataset) and multiple tissues [11]. In both studies, the difference between methylation-predicted age and chronological age (that is, Δ_age_) was put forth as an index of disproportionate ‘biological’ aging and was hypothesized to be associated with risk for age-related diseases and mortality [10,11]. Weidner *et al.* [12] proposed an age predictor based on three CpGs taken from a methylation array with fewer total CpG sites than the Hannum and Horvath models (27 k probes versus 450 k probes). To date, however, no study has tested whether DNA methylation-based Δ_age_ or other genome-wide DNA methylation biomarkers are significant predictors of all-cause mortality.

Here, we tested the association of two DNA methylation measures of Δ_age_ (using the Hannum and Horvath predictors) with all-cause mortality in four cohorts: the Lothian Birth Cohorts of 1921, and 1936 [13-15], the Framingham Heart Study [16,17], and the Normative Aging Study [18,19]. In addition, we estimated the heritability of Δ_age_ using the Brisbane Systems Genetics Study (BSGS) [20].

## Results

**Figure 1:**
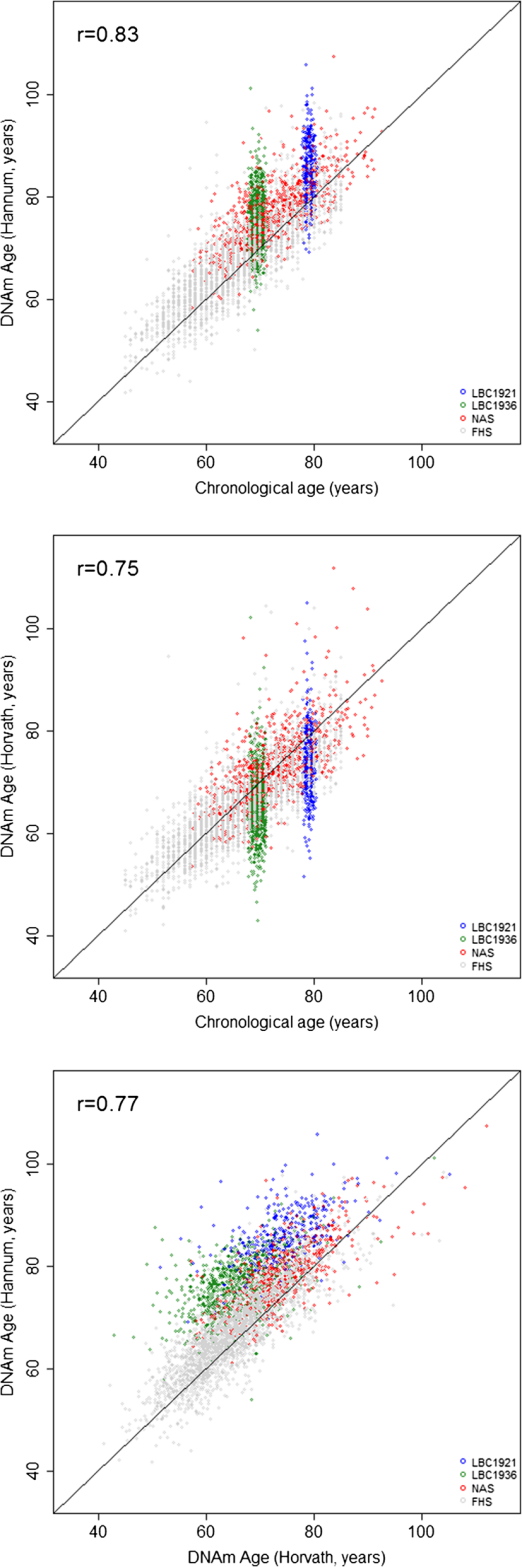
**Plot of predicted methylation age against chronological age and plot of Hannum versus Horvath predicted methylation age.** *To prevent the potential identification of individual participants, only FHS data points with chronological ages between 45 and 85, and NAS data points between ages 56 and 100 are displayed. r = Pearson correlation coefficient. FHS: Framingham Heart Study, LBC: Lothian Birth Cohort, NAS: Normative Aging Study.

The association between Δ_age_ (DNA methylation-predicted age minus chronological age) and mortality was examined in four cohorts: Lothian Birth Cohort 1921 (LBC1921) (N = 446, n_deaths_ = 292), Lothian Birth Cohort 1936 (LBC1936) (N = 920, n_deaths_ = 106), the Framingham Heart Study (FHS) (N = 2,635, n_deaths_ = 238), and the Normative Aging Study (NAS) (N = 657, n_deaths_ = 226). The mean ages of the cohorts were 79.1 (SD 0.6), 69.5 (SD 0.8), 66.3 (SD 8.9), and 72.9 (SD 6.9) years, respectively. The Hannum predicted values were higher than the participants’ chronological ages by a mean of 2 to 6 years (SDs approximately 5 years) across the four cohorts. The Horvath predicted values were lower than the chronological ages in LBC1921 and LBC1936 participants by 4 to 5 years (SD approximately 6 years) but very similar to chronological age in the FHS (−0.60 years; SD 5.2) and the NAS (0.6 years; SD 5.8). A third predictor, based on the Weidner predictor was also examined, although it had a low correlation with chronological age (LBC1921: Pearson R = 0.02; LBC1936: Pearson R = −0.03; FHS: Pearson R = 0.25; NAS: Pearson R = 0.43) and very large absolute median differences (LBC1921: 29.9 years, LBC1936: 19.8 years, FHS: 12.6 years, NAS: 18.4 years) so was not examined further. A full description of the cohorts is provided in Table 1 and Additional file 1. Combining information from these studies, the correlation between chronological age and predicted age was 0.83 for the Hannum measure and 0.75 for the Horvath measure (Figure 1). The correlation between the Hannum and Horvath predictors was 0.77.

**Table 1:** **Summary details of the four analysis cohorts**

	**LBC1921**	**LBC1936**	**FHS**	**NAS**
N	446	920	2,635	657
n_deaths_	292	106	238	226
Time to death (years)	7.2 (3.5)	4.4 (2.2)	6.0 (1.2)	10.5 (3.3)
Age (years)	79.1 (0.6)	69.5 (0.8)	66.3 (8.9)	72.9 (6.9)
Sex (male)	176 (40%)	465 (51%)	1200 (46%)	657 (100%)
Hannum methylation age (years)	85.0 (5.6)	75.8 (5.0)	68.2 (8.7)	77.6 (6.7)
Hannum Δ_age_ (years)	5.9 (5.6)	6.2 (5.1)	1.9 (4.8)	4.6 (4.8)
Hannum median error (years)	5.5	6.4	1.9	4.6
Horvath methylation age (years)	73.7 (6.2)	66.0 (5.8)	65.7 (8.3)	73.5 (7.4)
Horvath Δ_age_ (years)	−5.4 (6.3)	−3.6 (5.8)	−0.6 (5.2)	0.6 (5.8)
Horvath median error (years)	6.0	4.7	0.69	3.4
Tissue sample	Whole blood	Whole blood	Whole blood	Buffy coat
Methylation array	Illumina 450 k	Illumina 450 k	Illumina 450 k	Illumina 450 k

FHS: Framingham Heart Study, LBC: Lothian Birth Cohort, NAS: Normative Aging Study.

### Methylation age acceleration predicts mortality

**Figure 2:**
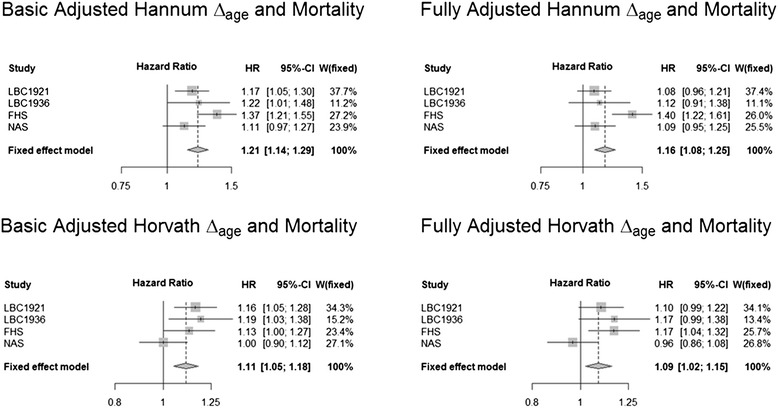
**Meta-analysis results of Δ**_**age**_ **versus mortality.** The basic adjusted models controlled for chronological age, sex (NAS had only male participants), and laboratory batch (FHS only). The fully adjusted models controlled for chronological age, sex, smoking, education, childhood IQ (LBC1921 and LBC1936 only), social class (LBC1921 and LBC1936 only), *APOE* (LBC1921, LBC1936, and NAS only), cardiovascular disease, high blood pressure, and diabetes. CI: confidence interval, FHS: Framingham Heart Study, HR: hazard ratio, LBC: Lothian Birth Cohort, NAS: Normative Aging Study, W: fixed effect weight.

**Figure 3:**
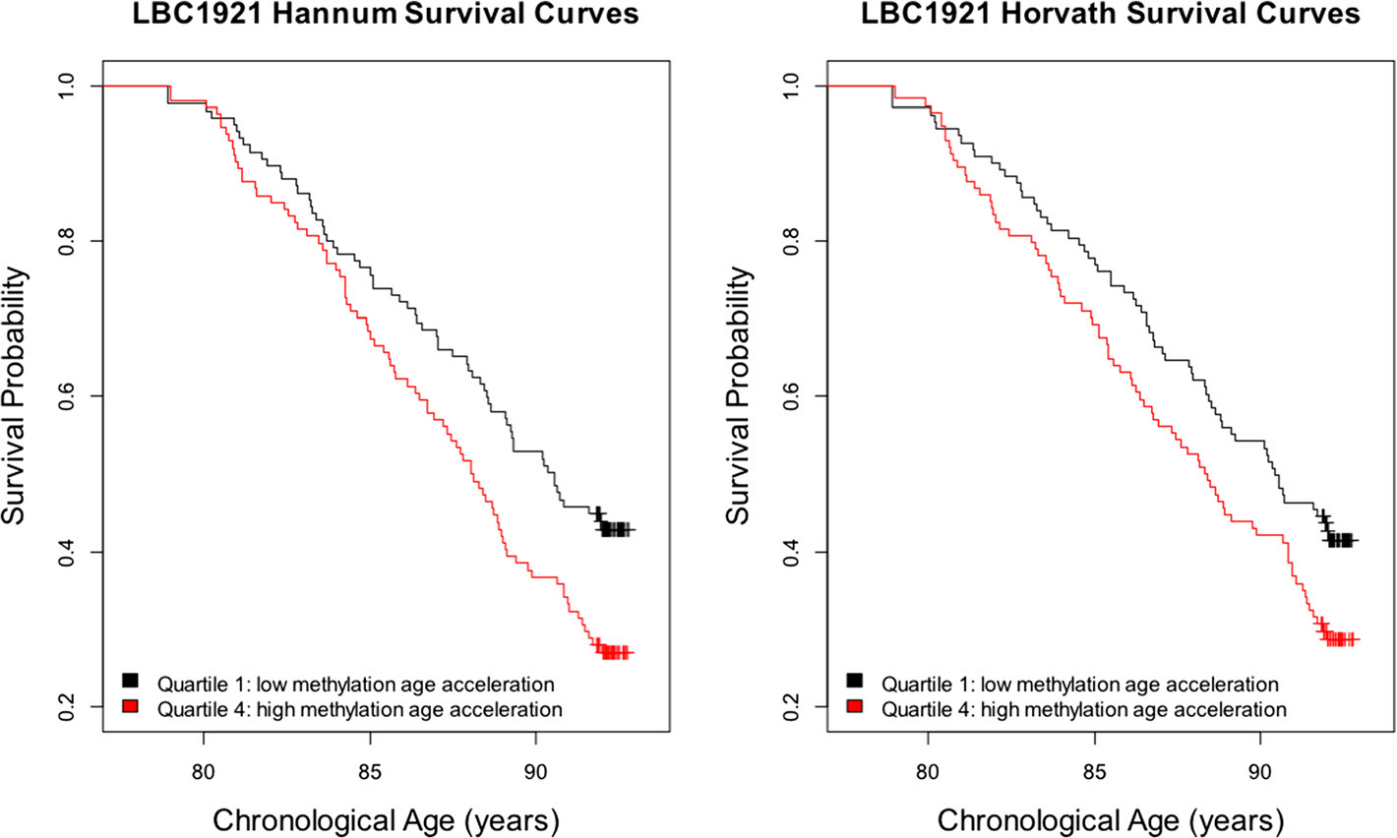
**Survival probability by quartiles of Δ**_**age**_ **in LBC1921 adjusted for sex, and chronological age.** LBC: Lothian Birth Cohort.

In the meta-analyzed results across the four cohorts, a 5-year higher Hannum Δ_age_ was associated with a 21% (95% CI (1.14, 1.29), *P* <0.0001) greater mortality risk after adjustment for chronological age and sex (Figure 2). The corresponding increase in mortality risk for the Horvath Δ_age_ was 11% (95% CI (1.05, 1.18), *P* = 0.0003). Kaplan-Meier survival curves for the Horvath and Hannum Δ_age_ (split into highest versus lowest quartile, for descriptive purposes only) in these models are presented in Figure 3 for the LBC1921 sample, which was the study with the greatest number of deaths. The plot illustrates the higher mortality rate for those with higher Δ_age_.

### Association between methylation age indicators and white blood cell counts

It is well known that blood cell types have distinct methylation profiles [21,22]. A sensitivity analysis adjusting for white blood cell counts (basophils, eosinophils, monocytes, lymphocytes, and neutrophils) resulted in mostly minor differences to the results (Additional file 2).

In addition to the five white blood cell types, we also examined the association between estimated naive T cell abundance and Δ_age_. However, to prevent spurious correlations between Δ_age_, which by definition correlates negatively with age, and cell counts, we used age acceleration defined as the residuals from a regression of predicted age on chronological age. There were stronger associations with these measures and the Hannum predictor (naive CD4+ T cells, average correlation r = −0.35, naive CD8+ T cells, average r = −0.34) compared to the Horvath predictor (r = −0.20, and −0.20, respectively). After adjustment for naive T cells, both predictors were still significantly associated with mortality. Naive T cell count was also associated with mortality in addition to the Hannum predictor (Additional file 3). Chronological age had a significant negative relationship with the abundance of naive T cells (on average r = −0.12 for CD8+ and r = −0.10 for CD4+ T cells, Additional file 3).

The moderately strong correlation between naive T cell abundance and the Hannum predictor suggests that the latter keeps track of the age-related decline of certain T cell populations. This makes sense in light of the facts that: (1) the Hannum predictor was constructed on the basis of DNA methylation data from whole blood; and (2) that naive T cells diminish with age due to age related thymic involution. In contrast to the Hannum predictor, the Horvath predictor exhibits a weaker relationship with naive T cell abundance, which probably reflects the fact that it was constructed on a range of different tissues and cell types.

### Adjusting for demographic variables and risk factors

Sensitivity analyses were performed to control for potentially confounding variables: smoking, education, childhood IQ (LBC1921 and LBC1936 only), social class (LBC1921 and LBC1936 only), *APOE* (LBC1921, LBC1936, and NAS only), cardiovascular disease, high blood pressure, and diabetes. When entered together in a fully adjusted model (Figure 2) the meta-analyzed hazards ratio (HR) per 5-year increment was 1.16 (95% CI (1.08, 1.25), P = 6.0x10^-9^) for the fully adjusted Hannum Δ_age_ and 1.09 (95% CI (1.02, 1.15), *P* = 0.0069) for the fully adjusted Horvath Δ_age_. In the LBC datasets, which were the only datasets to contain information on all of the covariates, inclusion of the covariates one at a time made very little difference to basic, age-, and sex-adjusted results (Additional file 4).

Separate age-adjusted Cox models for men and women are presented in Additional file 5; there was no notable difference in the relation of Δ_age_ to survival by sex. Sensitivity analyses that excluded deaths within the first 2 years of follow-up made negligible differences to the effect size and significance of the Δ_age_ associations (Additional file 6).

We tested the associations between Δ_age_ and several key covariates (Additional file 7). With the exception of sex, where women had significantly lower Δ_age_ estimates than men, there were no consistent associations with the covariates. There was some evidence for an association between the Hannum (but not Horvath) Δ_age_ and childhood IQ and social class, although these covariates were assessed in only the two LBC cohorts.

### Heritability of methylation Δ_age_

**Figure 4:**
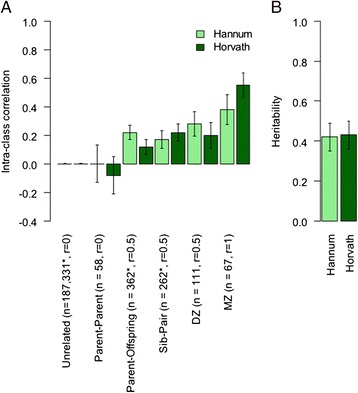
**Heritability of methylation Δ**_**age**_ **. (A)** Intra-class correlation of Hannum and Horvath Δ_age_ across relationship class. **(B)** Heritability of Hannum and Horvath Δ_age_ in BSGS data. Both plots show estimates with standard errors. *Pseudo-independent pairs. r represents the degree of relatedness.

Using data from the BSGS cohort, the estimated heritability for the Horvath and Hannum Δ_age_ was 0.43 (SE 0.07, *P* = 9×10^−13^) and 0.42 (SE 0.07, *P* = 4×10^−10^), respectively, indicating that approximately 40% of inter-individual differences in Δ_age_ are due to genetic factors. The contribution to heritability broken down across relationship classes is given in Figure 4, and is consistent with an additive genetic model of inheritance.

## Discussion

The difference between DNA methylation predicted age and chronological age (Δ_age_) using two sets of epigenetic markers [10,11] is a heritable trait that is associated with an increased risk of mortality in four independent cohorts of older individuals. This association is independent of life-course predictors of aging and death such as possession of the e4 allele of *APOE*, education, childhood IQ, social class, diabetes, high blood pressure, and cardiovascular disease. Moreover, there was no clear association between these factors and Δ_age_.

A strength of our study is that we evaluate two distinct epigenetic biomarkers of aging (Δ_age_) one from DNA methylation in whole blood, and one based on results across multiple tissues. The associations between Δ_age_ and mortality were stronger for the blood-based predictor but the two measures became comparable after adjusting for naive CD8 T cell abundances (Additional file 3). It is well known that immunosenescence is accompanied by diminishing naive T cells due to thymic involution and that the aging immune system is a predictor of human longevity [23,24]. While it is possible that the association between accelerated epigenetic age and all-cause mortality is mediated by changes in blood cell composition, we think that this is unlikely for the following reasons. First, the Horvath measure only exhibited a weak relationship (average r = −0.20) with naive T cell abundance which reflects its definition and applicability to most tissues and cell types. Second, both measures remained significant predictors of mortality in multivariate regression models that adjusted for naive T cell abundances.

Given the similarity of the findings for the two epigenetic biomarkers of aging, it is a credible hypothesis that Δ_age_ is an epigenetic biomarker of the pace of biological aging throughout life.

Nearly all biological and lifestyle factors that we studied did not materially influence either the Hannum or Horvath Δ_age_ across the four cohorts. Specifically, vascular insults, as measured by diabetes and hypertension, which are linked to cognitive decline, dementia, and death [25-27], were not associated with Δ_age_. There were sex differences, with men having higher Δ_age_ than women, which is consistent with previous findings [10]. Childhood IQ and social class were modestly associated with Δ_age_ in the two Lothian Birth Cohorts; an increased IQ in childhood and a less deprived social class were associated with lower Δ_age_ in later life, although these were driven almost entirely by LBC1936. These correlated variables have been repeatedly associated with a range of health inequalities, including mortality [28], and it is possible that Δ_age_ might offer insight into the mechanisms by which they are linked to health outcomes.

## Conclusions

There is continued interest in identifying new risk factors, environmental, genetic, and epigenetic that can improve our ability to predict disease and mortality. Epidemiological studies have identified numerous measures from across the human life-course that are associated with an increased risk of mortality. These include health factors such as cardiovascular disease, diabetes, and hypertension [27], genetic factors such as presence of the *APOE* e4 allele [29], lifestyle variables such as smoking [30] and education [31], behavioral traits such as cognitive ability [31,32], the personality trait of conscientiousness [33], and candidate biomarkers of age such as telomere length [34,35]. Here, we report on an epigenetic biomarker that is predictive of human mortality, after accounting for known risk factors. We found that two heritable DNA methylation-based measures of the difference between epigenetic age and chronological age are significant predictors of mortality in our meta-analysis of four independent cohorts of older people. Individual genetic or environmental exposures that drive the associations are not yet known, but they appear not to be clearly linked to classic life-course risk factors. The difference between DNA methylation age and chronological age predicts mortality risk over and above a combination of smoking, education, childhood IQ, social class, *APOE* genotype, cardiovascular disease, high blood pressure, and diabetes. It may therefore be possible to think of DNA methylation predicted age as an ‘epigenetic clock’ [11] that measures biological age and runs alongside, but not always in parallel with chronological age, and may inform life expectancy predictions. Our results imply that epigenetic marks, such as gene methylation, are like other complex traits: influenced by both genetic and environmental factors and associated with major health-related outcomes.

## Materials and methods

### Cohort descriptions

#### The Lothian Birth Cohort 1921

Data were from the Lothian Birth Cohort 1921 (LBC1921), which is the basis of a longitudinal study of aging [13,15]. Participants were born in 1921 and most completed a cognitive ability test at about the age of 11 years in the Scottish Mental Survey 1932 (SMS1932) [36]. The SMS1932 was administered nationwide to almost all 1921-born children who attended school in Scotland on 1 June 1932. The cognitive test was the Moray House Test No. 12, which provides a measure of general cognitive ability and has a scoring range between 0 and 76. The LBC1921 study attempted to follow up individuals who might have completed the SMS1932 and resided at about the age of 79 years in the Lothian region (Edinburgh and its surrounding areas) of Scotland; 550 people (n = 234, 43% men) were successfully traced and participated in the study from the age of 79 years. To date, there have been four additional follow-up waves at average ages of 83, 87, 90, and 92 years. The cohort has been deeply phenotyped during the later-life waves, including blood biomarkers, cognitive testing, and psycho-social, lifestyle, and health measures [13]. Genome wide single nucleotide polymorphisms and exome chip data are also available. DNA methylation measured in subjects at an average age of 79 (n = 514) was used for analyses in this report.

#### Lothian Birth Cohort 1936

The methylation mortality survival analysis was investigated in a second study, the Lothian Birth Cohort 1936 (LBC1936) [13,14]. All participants were born in 1936. Most had taken part in the Scottish Mental Survey 1947 at a mean age of 11 years as part of national testing of almost all children born in 1936 who attended Scottish schools on 4 June 1947 [37]. The cognitive test administered was the same Moray House Test No. 12 used in the SMS1932. A total of 1,091 participants (n = 548, 50% men) who were living in the Lothian area of Scotland were re-contacted in later life. Extensive phenotyping has also been carried out in this study, with data collection waves at three time points [13]. Genome-wide single nucleotide polymorphisms and exome chip data are also available. DNA methylation was measured in 1,004 subjects at Wave 1 (mean age, 70 years). To date, there have been two additional follow-up waves at average ages of 73 and 76 years.

#### The Framingham Heart Study

Framingham Heart Study (FHS) is a community-based longitudinal study of participants living in and near Framingham, MA, at the start of the study in 1948 [16]. The Offspring cohort comprised the children and spouses of the original FHS participants, as described previously [17]. Briefly, enrollment for the Offspring cohort began in 1971 (n = 5,124), and in-person evaluations occurred approximately every 4 to 8 years thereafter. The current analysis was limited to participants from the Offspring cohort who survived until the eighth examination cycle (2005 to 2008) and consented to genetics research. DNA methylation data of peripheral blood samples collected at the eighth examination cycle were available in 2,741 participants.

#### The Normative Aging Study

The US Department of Veterans Affairs (VA) Normative Aging Study (NAS) is an ongoing longitudinal cohort established in 1963, which included men who were aged 21 to 80 years and free of known chronic medical conditions at entry [18,19]. Participants were subsequently invited to medical examinations every 3 to 5 years. At each visit, participants provided information on medical history, lifestyle, and demographic factors, and underwent a physical examination and laboratory tests. DNA samples were collected from 1999 to 2007 from the 675 active participants and used for DNA methylation analysis. We excluded 18 participants who were not of European descent or had missing information on race, leaving a total of 657 individuals.

#### Brisbane Systems Genetics Study

The Brisbane Systems Genetic Study (BSGS) [20] is a cohort comprising adolescent monozygotic (MZ) and dizygotic (DZ) twins, their siblings, and their parents. They were originally recruited into an ongoing study of the genetic and environmental factors influencing cognition and pigmented nevi. DNA methylation was measured on 614 individuals from 117 families of European descent. Families consist of adolescent monozygotic (MZ; n = 67 pairs) and dizygotic (DZ; n = 111 pairs) twins, their siblings (n = 119), and their parents (n = 139). Children have a mean age of 14 years (age range, 9–23 years) and parents 47 years (age range, 33–75 years).

### Ethics

#### LBC consent

Following informed consent, venesected whole blood was collected for DNA extraction in both LBC1921 and LBC1936. Ethics permission for the LBC1921 was obtained from the Lothian Research Ethics Committee (Wave 1: LREC/1998/4/183). Ethics permission for the LBC1936 was obtained from the Multi-Centre Research Ethics Committee for Scotland (Wave 1: MREC/01/0/56), the Lothian Research Ethics Committee (Wave 1: LREC/2003/2/29). Written informed consent was obtained from all subjects.

#### FHS consent

All participants provided written informed consent at the time of each examination visit. The study protocol was approved by the Institutional Review Board at Boston University Medical Center (Boston, MA, USA).

#### NAS consent

The NAS study was approved by the Institutional Review Boards (IRBs) of the participating institutions. Participants have provided written informed consent at each visit.

#### BSGS consent

The BSGS study was approved by the Queensland Institute for Medical Research Human Research Ethics Committee. All participants gave informed written consent.

### DNA methylation measurement

In all cohorts, bisulphite converted DNA samples were hybridised to the 12 sample Illumina HumanMethylation450BeadChips [38] using the Infinium HD Methylation protocol and Tecan robotics (Illumina, San Diego, CA, USA).

#### LBC1921 and LBC1936 DNA methylation

DNA was extracted from 514 whole blood samples in LBC1921 and from 1,004 samples in LBC1936. Samples were extracted at MRC Technology, Western General Hospital, Edinburgh (LBC1921) and the Wellcome Trust Clinical Research Facility (WTCRF), Western General Hospital, Edinburgh (LBC1936), using standard methods. Methylation typing of 485,512 probes was performed at the WTCRF. Raw intensity data were background-corrected and methylation beta-values generated using the R minfi package [39]. Quality control analysis was performed to remove probes with a low (<95%) detection rate at *P* <0.01. Manual inspection of the array control probe signals was used to identify and remove low quality samples (for example, samples with inadequate hybridization, bisulfite conversion, nucleotide extension, or staining signal). The Illumina-recommended threshold was used to eliminate samples with a low call rate (samples with <450,000 probes detected at *P* <0.01). Since the LBC samples had previously been genotyped using the Illumina 610-Quadv1 genotyping platform, genotypes derived from the 65 SNP control probes on the methylation array using the wateRmelon package [40] were compared to those obtained from the genotyping array to ensure sample integrity. Samples with a low match of genotypes with SNP control probes, which could indicate sample contamination or mix-up, were excluded (n = 9). Moreover, eight subjects whose predicted sex, based on XY probes, did not match reported sex were also excluded.

#### FHS DNA methylation

Peripheral blood samples were collected at the eighth examination samples (2005 to 2008). Genomic DNA was extracted from buffy coat using the Gentra Puregene DNA extraction kit (Qiagen) and bisulfite converted using EZ DNA Methylation kit (Zymo Research Corporation). DNA methylation quantification was conducted in two laboratory batches. Methylation beta values were generated using the Bioconductor minfi package with background correction. Sample exclusion criteria included poor SNP matching of control positions, missing rate >1%, outliers from multi-dimensional scaling (MDS), and sex mismatch. Probes were excluded if missing rate >20%. In total, 2,635 samples and 443,304 CpG probes remained for analysis.

#### NAS DNA methylation

DNA was extracted from buffy coat using the QIAamp DNA Blood Kit (QIAGEN, Valencia, CA, USA). A total of 500 ng of DNA was used to perform bisulfite conversion using the EZ-96 DNA Methylation Kit (Zymo Research, Orange, CA, USA). To limit chip and plate effects, a two-stage age-stratified algorithm was used to randomize samples and ensure similar age distributions across chips and plates; we randomized 12 samples - which were sampled across all the age quartiles - to each chip, then chips were randomized to plates (each housing eight chips). Quality control analysis was performed to remove samples where >1% of probes had a detection *P* value >0.05. The remaining samples were preprocessed using the Illumina-type background correction without normalization as reimplemented in the Bioconductor minfi package, which was used to generate methylation beta values [39]. All 485,512 CpG and CpH probes were in the working set.

#### BSGS DNA methylation

DNA was extracted from peripheral blood lymphocytes by the salt precipitation method [41] from samples that were time matched to sample collection of PAXgene tubes for gene expression studies in the Brisbane Systems Genetics Study [20]. Bisulphite converted DNA samples were hybridized to the 12 sample Illumina HumanMethylation450 BeadChips using the Infinium HD Methylation protocol and Tecan robotics (Illumina, San Diego, CA, USA). Samples were randomly placed with respect to the chip they were measured on and to the position on that chip in order to avoid any confounding with family. Box-plots of the red and green intensity levels and their ratio were used to ensure that no chip position was under- or over-exposed, with any outlying samples repeated. Similarly, the proportion of probes with detection *P* value less than 0.01 was examined to confirm strong binding of the sample to the array. Raw intensity values were background corrected using the Genome Studio software performing normalization to internal controls and background subtraction.

### Mortality ascertainment

#### LBC mortality ascertainment

For both LBC1921 and LBC1936, mortality status was obtained via data linkage from the National Health Service Central Register, provided by the General Register Office for Scotland (now National Records of Scotland). Participant deaths and cause of death are routinely flagged to the research team on approximately a 12-weekly basis.

#### FHS mortality ascertainment

Deaths that occurred prior to 1 January 2013 were ascertained using multiple strategies, including routine contact with participants for health history updates, surveillance at the local hospital and in obituaries of the local newspaper, and queries to the National Death Index. We requested death certificates, hospital and nursing home records prior to death, and autopsy reports. When cause of death was undeterminable, the next of kin were interviewed. The date and cause of death were reviewed by an endpoint panel of three investigators.

#### NAS mortality ascertainment

Regular mailings to study participants have been used to maintain vital-status information, and official death certificates were obtained for decedents from the appropriate state health department. Death certificates were reviewed by a physician, and cause of death coded by an experienced research nurse using ICD-9. Both participant deaths and cause of death are routinely updated by the research team and last update available was 31 December 2013.

### Covariate measurement

#### LBC covariates

Mortality-associated variables assessed in LBC1921 and LBC1936 were used as covariates in the statistical models: educational attainment, age-11 cognitive ability, *APOE* e4 status (carriers versus non-carriers), smoking status, and the presence or absence of diabetes, high blood pressure, or cardiovascular disease. Age-11 cognitive ability (age-11 IQ) was measured in 1932 for LBC1921 and in 1947 for LBC1936 using the Moray House Test Number 12, described above. All other variables were measured at the late-life baseline waves (age 79 years for LBC1921 and age 70 years for LBC1936). *APOE* was genotyped from venous blood using PCR amplification of a 227-bp fragment of the *APOE* gene, which contains the two single nucleotide polymorphisms that are used to define the e2, e3, and e4 alleles [42] in LBC1921, and by TaqMan technology (Applied Biosystems, Carlsbad, CA, USA) in LBC1936. Subjects were then categorized by the presence or absence of the e4 allele. Social class was based on the most prestigious occupation held by the participant prior to retirement. It was grouped into five categories in LBC1921 and six categories in LBC1936, where Class III was split into manual and non-manual professions [43,44]. It was treated as a continuous variable with lower values representing the more prestigious classes. The other variables were determined via self-report: number of years of education (measured as a continuous variable), diabetes (yes/no), high blood pressure (yes/no), cardiovascular disease (yes/no), and categorical smoking status (current/ex-smoker, never smoked).

Given the known influence of blood cell count on methylation [21], we adjusted for five types of white blood cell count (basophils, monocytes, lymphocytes, eosinophils, and neutrophils) that were measured at on the same blood that was analyzed for methylation. These data were collected and processed the same day; technical details are reported in McIllhagger *et al*. [45].

#### FHS covariates

At the eighth in-person examination visit participants completed a questionnaire that inquired about their education, occupation, smoking status, and disease status. Highest levels of educational attainment was assessed by eight categories - no schooling, grades 1 to 8, grades 9 to 11, completed high school or GED, some college but no degree, technical school certificate, associate degree, Bachelor’s degree, graduate or professional degree. Smoking status was dichotomized as current/past smokers and those who reported to never have smoked. Diabetes was defined as having fasting blood glucose ≥126 mg/dl or current treatment for diabetes. Hypertension was defined as having systolic blood pressure ≥140 mmHg, diastolic blood pressure ≥90 mmHg, or current treatment for hypertension. Cardiovascular disease was determined by a panel of three physicians, who reviewed participants’ medical records, laboratory findings, and clinic exam notes.

#### NAS covariates

At each in-person examination visit, participants completed a questionnaire that enquired about their smoking status, education, diabetes (self-reported diagnosis and/or use of diabetes medications), and diagnosis of coronary heart disease (validated on medical records, ECG, and physician exams). High blood pressure was defined as antihypertensive medication use or SBP ≥140 mmHg or DBP ≥90 mmHg at study visit. *APOE*-e4 allele status was assessed through genotyping on a Sequenom MassArray MALDI-TOF mass spectrometer.

#### Estimated naive T cell abundance

In LBC1921, LBC1936, FHS, and NAS, we considered the abundance of defined different subtypes of T cells: Naive T cells were defined as RA+ IL7 Receptor + cells. Central Memory T cells = RA negative IL7 Receptor positive Effector memory = RA negative IL7 Receptor negative. To estimate the naive T cells in our cohort studies, we used a prediction method that was developed on an independent dataset. The predictor of T cell counts (that is, naive CD4 T cell count) was found by applying a penalized regression model (elastic net) to regress T cell counts (dependent variable) on a subset of CpGs reported in Supplemental Table 3 from Zhang *et al*. [46]. By applying this resulting penalized regression model to our data, we arrived at predicted T cell counts.

#### Data availability

LBC methylation data have been submitted to the European Genome-phenome Archive under accession number EGAS00001000910; phenotypic data are available at dbGaP under the accession number phs000821.v1.p1. The FHS and NAS data are available at dbGaP under the accession numbers phs000724.v2.p9 phs000853.v1.p1, respectively. BSGS methylation data are available from the NCBI Gene Expression Omnibus under accession number GSE56105.

### Statistical analyses

Two measures of DNA methylation age (m_age_) were calculated. The Horvath [11] m_age_ uses 353 probes common to the Illumina 27 K and 450 K Methylation arrays using data from a range of tissues and cell types. The Hannum [10] m_age_ is based on 71 methylation probes from the Illumina 450 K Methylation array derived as the best predictors of age using data generated from whole blood. Of the Hannum age predictor probes, 70, 71, and 71 were included in the LBC, NAS, and FHS data, respectively. m_age_ was calculated as the sum of the beta values multiplied by the reported effect sizes for the Hannum predictor. For the Horvath predictor, m_age_ was determined in all cohorts using the online calculator (http://labs.genetics.ucla.edu/horvath/dnamage/). A third predictor, based on the three probes highlighted in the Weidner *et al.* paper [12], was also examined although, due to its poorer predictive accuracy, it was not included for the main analyses. To account for technical variability in the measurement of the methylation CpGs in the LBC studies, m_age_ was adjusted for plate, array, position on the chip, and hybridisation date (all treated as fixed effect factors) using linear regression. In a sensitivity analysis, additional adjustments were made for white blood cell counts (the number of basophils, monocytes, lymphocytes, eosinophils, and neutrophils per volume of blood) or DNA methylation-estimated cell counts, as described elsewhere [21]. The residuals from these models were added to the mean predicted methylation age to give the new, adjusted measure of m_age_. The two methylation age predictors contained six overlapping probes. A methylation-based age acceleration index (Δ_age_) was calculated for all subjects, defined as the adjusted methylation age in years minus chronological age at sample collection in years (Δ_age_ = m_age_ - chronological age).

Cox proportional hazards regression models were used to test the association between the Horvath and Hannum measures of Δ_age_ and mortality, adjusting for age at sample collection, and sex. Cox models in FHS further adjusted for laboratory batch (fixed effect) and used a robust variance estimator to account for familial relatedness. Hazard ratios for Δ_age_ were expressed per 5 years of methylation age acceleration. Schoenfeld residuals were examined to test the proportional hazards assumption. Sensitivity analyses, also using Cox proportional hazards regression, excluded deaths within the first 2 years of follow-up to eliminate the potential influences of (fatal) acute illness on the methylation measurements. Analyses to account for possible confounders/mediators included potential life-course predictors of mortality: age-11 IQ (LBC only), education in years, social class (LBC only), *APOE* e4 carrier status (LBC and NAS), smoking status, and self-reported diabetes, high blood pressure, and cardiovascular disease. A fully adjusted model was tested, in which all variables were entered together. Chronological age- and sex-adjusted linear regression models were used to explore the relationship between Δ_age_ and the additional covariates; for example, does methylation age acceleration depend on smoking or diabetes?

The results from the individual cohorts were meta-analyzed using the ‘meta’ package in R [47]. The cohorts were weighted based on the standard errors of the log hazard ratios. There was no evidence of cohort heterogeneity in the primary Cox model analyses according to the DerSimonian-Laird estimator of between-study variance so fixed effects models were considered.

All analyses were performed in the statistical software R [48] with the Cox models utilizing the 'survival' library [49].

Finally, we calculated the heritability of Δ_age_ in the BSGS cohort. As m_age_ was a better predictor of chronological age in the adult compared to adolescent samples, the difference between methylation age and chronological age was firstly standardized within generations (parents and offspring). Regression models were fitted to methylation age removing the effects of age and sex. Additionally, the regression on the adolescent samples included age^2^ to account for the non-linearity between chronological and methylation age [11]. The residuals from these regressions were standardized to have a variance of 1 before combining the generations. See Additional file 8 for a graphical representation of the correction performed.

For each probe, the Intra Class Correlation of Δ_age_ for the various relative pairs was calculated using ANOVA as follows:

$$ ICC=\frac{M{S}_B-M{S}_W}{M{S}_B+M{S}_W} $$

where *MS*_*B*_ is the Mean Square Between pairs and *MS*_*W*_ is the Mean Square Within. The confidence intervals were based on the number of pseudo-independent relative pair for each relationship.

The heritability for each probe was estimated by partitioning its variance into additive genetic (*V*_*a*_) and environmental (*V*_*e*_) component by fitting a linear mixed model of the form

$$ \mathbf{y}=\mu +\mathbf{Z}\mathbf{a}+\mathbf{e} $$

where **y** is the vector of adjusted methylation age, **a** is the additive genetic effects and **e** is the unique environmental effects (residuals). The model was fitted using QTDT [50].

## Additional files

Additional file 1:
**Contains a table with summary information for the additional covariate data in the four cohorts.**
Additional file 2:
**Contains a table with the white blood cell-adjusted associations of Horvath and Hannum Δ**_**age**_ **with mortality.**
Additional file 3:
**Presents the results from the analyses that accounted for differences in naive T cell abundance.** It contains a table and cohort specific figures that assess the association between naive T cell abundance and age acceleration, chronological age, and mortality. Cox model output is also included to show the association between methylation age acceleration and mortality after adjusting for naive T cells. Additional file 4:
**Contains a table of the associations of Δ**_**age**_ **(per 5 years) with mortality in LBC1921 and LBC1936 after individual adjustment for covariates.** The basic adjustment model controls for age and sex. A separate Cox model adjusting for age, sex, and a single covariate was analyzed along with a saturated model that included age, sex, and all covariates together. Additional file 5:
**Contains a figure with the meta-analysis results of sex-stratified, age-adjusted models of Δ**_**age**_ **against mortality.**
Additional file 6:
**Contains a figure with the meta-analysis results of age- and sex-adjusted Δ**_**age**_ **against mortality with a 2-year time lag.**
Additional file 7:
**Contains a table with the associations of Δ**_**age**_ **with known mortality risk factors.** Separate linear regression analyses were performed for each covariate. All models adjusted for sex except for NAS, which only had male participants. Analysis of FHS data was adjusted for laboratory batch and family structure. Additional file 8:
**Contains a figure illustrating the model fitting in BSGS to create residuals from a regression of predicted age against chronological age.** A quadratic chronological age term was included in the adolescent model along with chronological age and sex (red line). A linear model adjusting for age and sex was included in the adult model (blue line).
