# Supplementary material for: DNA methylation age of blood predicts all-cause mortality in later life
Source: Genome Biol. 2015 Jan 30;16(1):25. doi: 10.1186/s13059-015-0584-6 (PMC4350614; doi:10.1186/s13059-015-0584-6)
Supplement: Additional file 1: — Contains a table with summary information for the additional covariate data in the four cohorts. [file 13059_2015_584_MOESM1_ESM.docx]

Additional data file 1: Summary of additional covariate data.

|  | **LBC1921** | | | **LBC1936** | | | **NAS** | | | **FHS** | | |
| --- | --- | --- | --- | --- | --- | --- | --- | --- | --- | --- | --- | --- |
|  | **N** | **Mean** | **SD** | **N** | **Mean** | **SD** | **N** | **Mean** | **SD** | **N** | **Mean** | **SD** |
| Education (years)^*^ | 444 | 11.0 | 2.5 | 920 | 10.7 | 1.1 | 656 | 15.07 | 2.98 | * | * | * |
| Age-11 IQ (Moray House Test score) | 401 | 46.3 | 12.1 | 867 | 48.9 | 11.9 | - | - | - | - | - | - |
| Occupational social class^†^ | 445 | 2.2 | 0.89 | 901 | 2.40 | 0.93 | - | - | - | - | - | - |
| Basophil count (10^9^/L) | 429 | 0.02 | 0.04 | 913 | 0.04 | 0.04 | 639 | 0.04 | 0.03 | - | - | - |
| Eosiniphil count (10^9^/L) | 429 | 0.17 | 0.18 | 913 | 0.17 | 0.13 | 639 | 0.21 | 0.16 | - | - | - |
| Monocyte count (10^9^/L) | 429 | 0.54 | 0.18 | 913 | 0.53 | 0.19 | 639 | 0.54 | 0.16 | - | - | - |
| Lymphocyte count (10^9^/L) | 429 | 1.70 | 1.11 | 909 | 1.82 | 0.59 | 643 | 1.68 | 2.13 | - | - | - |
| Neutrophil count (10^9^/L) | 429 | 4.57 | 1.36 | 913 | 4.45 | 1.54 | 643 | 3.87 | 1.22 | - | - | - |
|  |  |  |  |  |  |  |  |  |  |  |  |  |
|  | **N** | **(n)** | **(%)** | **N** | **(n)** | **(%)** | **N** | **(n)** | **(%)** | **N** | **(n)** | **(%)** |
| Diabetes (yes) | 446 | 21 | 4.7 | 920 | 77 | 8.4 | 657 | 120 | 18.2 | 2623 | 362 | 13.8 |
| Cardiovascular Disease (yes) | 442 | 139 | 31.4 | 920 | 224 | 24.3 | 657 | 184 | 28.0 | 2628 | 419 | 15.9 |
| High Blood Pressure (yes) | 446 | 182 | 40.8 | 920 | 374 | 40.7 | 657 | 460 | 70.0 | 2627 | 1661 | 63.2 |
| *APOE* e4 carrier (yes) | 446 | 122 | 27.4 | 884 | 266 | 30.1 | 617 | 144 | 23.3 | - | - | - |
| Smoking | 445 |  |  | 920 |  |  | 657 |  |  | 2527 |  |  |
| Never |  | 201 | 45.2 |  | 432 | 47.0 |  | 467 | 71.1 |  | 898 | 35.5 |
| Ex or Current |  | 244 | 54.8 |  | 488 | 53.0 |  | 190 | 28.9 |  | 1629 | 64.5 |

LBC: Lothian Birth Cohort, NAS: Normative Aging Study, FHS: Framingham Heart Study, SD: Standard Deviation

^*^ FHS education was measured as an ordinal variable (no schooling: n=19 (1%), 1-8th grade: n=22 (1%), 9-11th grade: n=71 (3%), high school degree or GED: n=690 (27%), some college: n=487 (19%), technical school: n=98 (4%), associate's degree: n=235 (9%), bachelor's degree: n=529 (20%), graduate degree: n=477 (18%)). ^†^An ordinal variable from one to five in LBC1921 and one to six in LBC1936, with class 1 the most prestigious, professional occupations, class 5 or 6 the most manual, least prestigious. This variable was treated as a continuous scale for the analyses.
