# Supplementary material for: DNA methylation age of blood predicts all-cause mortality in later life
Source: Genome Biol. 2015 Jan 30;16(1):25. doi: 10.1186/s13059-015-0584-6 (PMC4350614; doi:10.1186/s13059-015-0584-6)
Supplement: Additional file 2: — Contains a table with the white blood cell-adjusted associations of Horvath and Hannum Δ age with mortality. [file 13059_2015_584_MOESM2_ESM.docx]

Additional data file 2: White blood cell-adjusted associations of Horvath and Hannum Δ_age_ with mortality.

|  | **Total N; n_deaths_** | **Hazard Ratio** | **95% CI** | **P** |
| --- | --- | --- | --- | --- |
| **LBC1921 Hannum** |  |  |  |  |
| Δ_age_ age- and sex-adjusted (per 5 years) | 429; 280 | 1.15 | [1.03, 1.28] | 0.01 |
| Δ_age_ fully-adjusted^*^ (per 5 years) | 376; 251 | 1.07 | [0.95, 1.21] | 0.26 |
|  |  |  |  |  |
| **LBC1921 Horvath** |  |  |  |  |
| Δ_age_ age- and sex-adjusted (per 5 years) | 429; 280 | 1.15 | [1.03, 1.27] | 9.6x10^-3^ |
| Δ_age_ fully-adjusted^*^ (per 5 years) | 376; 251 | 1.09 | [0.98, 1.22] | 0.13 |
|  |  |  |  |  |
| **LBC1936 Hannum** |  |  |  |  |
| Δ_age_ age- and sex-adjusted (per 5 years) | 909; 106 | 1.20 | [0.98, 1.46] | 0.08 |
| Δ_age_ fully-adjusted^*^ (per 5 years) | 805; 91 | 1.09 | [0.87, 1.35] | 0.45 |
|  |  |  |  |  |
| **LBC1936 Horvath** |  |  |  |  |
| Δ_age_ age- and sex-adjusted (per 5 years) | 909; 106 | 1.18 | [0.99, 1.39] | 0.06 |
| Δ_age_ fully-adjusted^*^ (per 5 years) | 805; 91 | 1.18 | [0.98, 1.42] | 0.08 |
|  |  |  |  |  |
| **FHS Hannum^†^** |  |  |  |  |
| Δ_age_ age- and sex-adjusted (per 5 years) | 2,448; 220 | 1.26 | [1.09, 1.46] | 0.002 |
| Δ_age_ fully-adjusted^*^ (per 5 years) | 2,361; 217 | 1.31 | [1.11, 1.54] | 0.001 |
|  |  |  |  |  |
| **FHS Horvath^†^** |  |  |  |  |
| Δ_age_ age- and sex-adjusted (per 5 years) | 2,449; 220 | 1.16 | [1.01, 1.33] | 0.03 |
| Δ_age_ fully-adjusted^*^ (per 5 years) | 2,362; 217 | 1.21 | [1.05, 1.39] | 0.008 |
|  |  |  |  |  |
| **NAS Hannum** |  |  |  |  |
| Δ_age_ age- and sex-adjusted (per 5 years) | 639; 221 | 1.17 | [1.00, 1.37] | 0.05 |
| Δ_age_ fully-adjusted^*^ (per 5 years) | 600; 212 | 1.15 | [0.98, 1.35] | 0.09 |
|  |  |  |  |  |
| **NAS Horvath** |  |  |  |  |
| Δ_age_ age-adjusted (per 5 years) | 639; 221 | 1.02 | [0.89, 1.17] | 0.76 |
| Δ_age_ fully-adjusted^*^ (per 5 years) | 600; 212 | 0.99 | [0.86, 1.14] | 0.86 |

LBC: Lothian Birth Cohort, FHS: Framingham Heart Study, NAS: Normative Aging Study, CI: Confidence Interval. ^*^adjusted for chronological age, sex (LBC and FHS cohorts only), smoking, education, age-11 IQ (LBC cohorts only), occupational social class (LBC cohorts only), *APOE* (LBC and NAS cohorts only), cardiovascular disease, high blood pressure, and diabetes. **^†^**Blood counts in FHS were estimated using the method described by Houseman EA, et al. (*BMC Bioinformatics.*2012; 13:86).
