# Supplementary material for: DNA methylation age of blood predicts all-cause mortality in later life
Source: Genome Biol. 2015 Jan 30;16(1):25. doi: 10.1186/s13059-015-0584-6 (PMC4350614; doi:10.1186/s13059-015-0584-6)
Supplement: Additional file 3: — Presents the results from the analyses that accounted for differences in naive T cell abundance. It contains a table and cohort specific figures that assess the association between naive T cell abundance and age acceleration, chronological age, and mortality. Cox model output is also included to show the association between methylation age acceleration and mortality after adjusting for naive T cells. [file 13059_2015_584_MOESM3_ESM.docx]

**Additional data file 3: Accounting for differences in naive T cell abundance**

Summary Table that summarizes the results from subsequent supplementary figures.

|  |  | **Pearson correlation coefficients** | | | | |
| --- | --- | --- | --- | --- | --- | --- |
| **Study Description** | **Measure** | **CD4+**  **naive** | **CD8+**  **naive** | **CD4+T** | **CD8+T** | |
| LBC 1921 | AA.Hannum | -0.38 | -0.37 | 0.00 | 0.03 | |
| LBC 1936 | AA.Hannum | -0.32 | -0.30 | -0.01 | -0.02 | |
| NAS | AA.Hannum | −0.30 | −0.37 | −0.26 | 0.16 | |
| FHS | AA.Hannum | -0.34 | -0.36 | -0.32 | -0.04 | |
|  | **AVERAGE** | **-0.35** | **-0.34** | **-0.11** | **0.03** | |
|  |  | **Pearson correlation coefficients** | | | | |
| LBC 1921 | AA.Horvath | -0.25 | -0.26 | -0.07 | 0.06 | |
| LBC 1936 | AA.Horvath | -0.16 | -0.13 | 0.00 | 0.01 | |
| NAS | AA.Horvath | −0.088 | −0.11 | −0.033 | 0.45 | |
| FHS | AA.Horvath | -0.18 | -0.20 | -0.20 | 0.15 | |
|  | **AVERAGE** | **-0.20** | **-0.20** | **-0.09** | **0.17** | |
|  |  | **Pearson correlation coefficients** | | | | |
| LBC 1921 | Chron. Age | -0.06 | -0.07 | 0.02 | -0.05 | |
| LBC 1936 | Chron. Age | 0.03 | 0.03 | -0.01 | -0.08 | |
| NAS | Chron. Age | −0.16 | −0.22 | −0.16 | 0.14 | |
| FHS | Chron. Age | -0.27 | -0.31 | -0.22 | -0.15 | |
|  | **AVERAGE** | **-0.10** | **-0.12** | **-0.07** | **-0.03** | |
|  |  |  |  |  |  | |
|  |  | **p-values (Kruskal Wallis test)** | | | | |
| LBC 1921 | Death | 1.2E-02 | 1.6E-03 | 2.8E-01 | | 5.4E-02 |
| LBC 1936 | Death | 4.9E-03 | 1.3E-03 | 5.3E-01 | | 5.2E-01 |
| NAS | Death | 9.4E-03 | 1.7e−07 | 8.4E-04 | | 7.2E-01 |
| FHS | Death | 3.9E-18 | 1.9E-24 | 1.4E-17 | | 1.2E-04 |
|  | **MEDIAN** | **7.2E-03** | **1.3E-03** | **1.4E-01** | | **2.9E-01** |

**Detailed results for LBC1921**


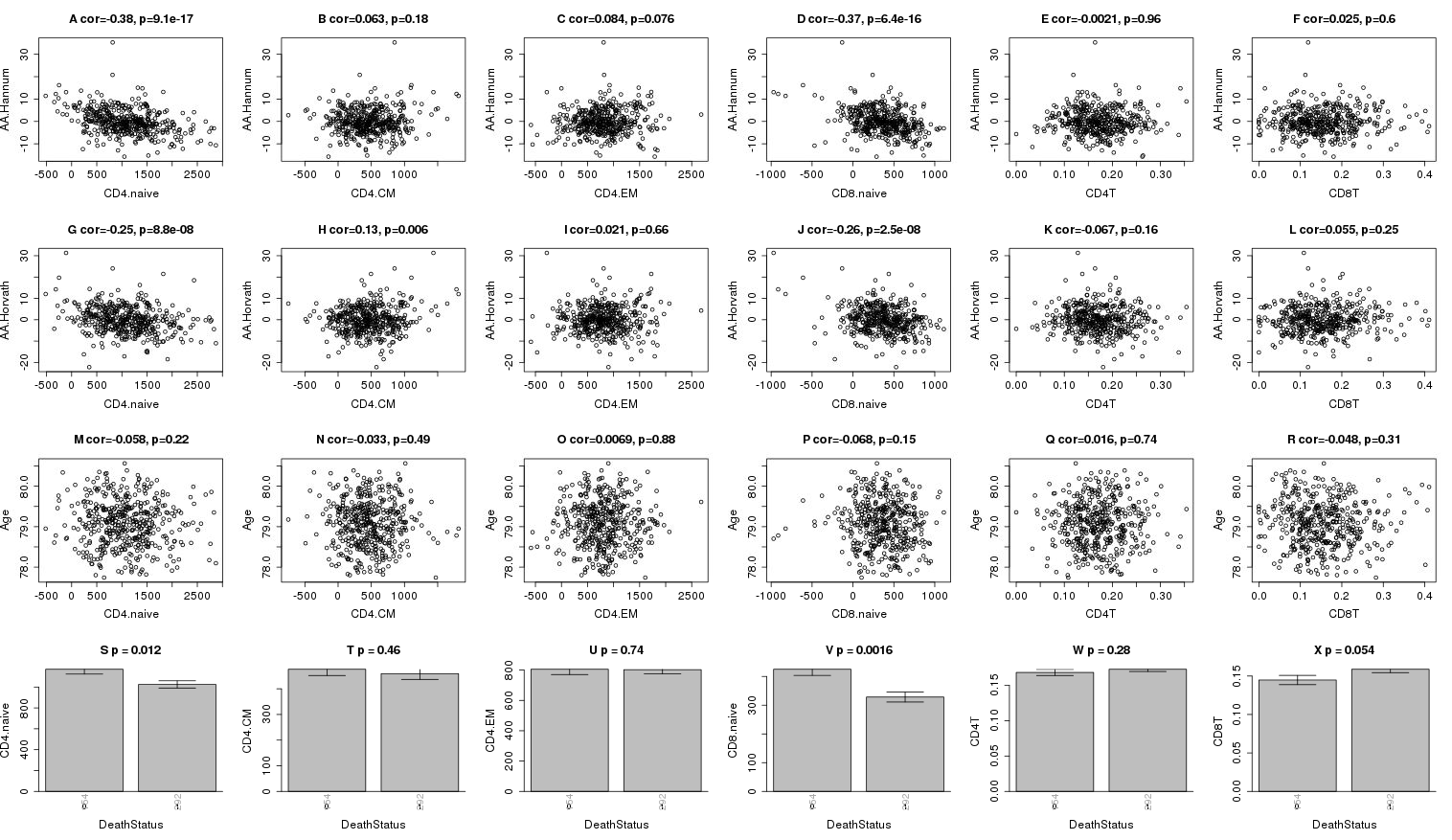


**LBC1921: T cell abundance measures versus age acceleration, chronological age and death status.**

A-F (first row): Age acceleration according to the Hannum predictor (y-axis) versus estimated abundance measures of naive CD4+ T cells (A), central memory CD4+T cells (B), effector memory CD4+T cells (C), naive CD8+ T cells (D), proportion of CD4+ T cells (E), and proportion of CD8+ T cells (F). The age acceleration measure was defined as residual resulting from regressing estimated age on chronological age. By definition, this age acceleration measure is uncorrelated with chronological age. G-L (second row) presents analogous results for the age predictor by Horvath. Note that it exhibits weaker correlations with naive T cell abundance than the age acceleration measure by Hannum. M-R (third row) analogous results for chronological age (y-axis). S-X (fourth row) relationship between death status (x-axis) and the various cell abundance measures. Each panel in the last row reports a non-parametric group comparison test (Kruskal Wallis test p-value). Each bar plot shows the mean value and 1 standard error.

Multivariate Cox regression analysis

coef exp(coef) se(coef) z Pr(>|z|)

DNAmAgeHorvath 0.0234121 1.0236883 0.0097840 2.393 0.016716 *

Age 0.0135687 1.0136612 0.1075160 0.126 0.899572

CD8.naive -0.0007115 0.9992887 0.0002120 -3.355 0.000792 ***

CD8T 1.4741953 4.3675200 1.3128354 1.123 0.261476

CD4T 2.3508566 10.4945553 1.6878646 1.393 0.163680

NK 1.3777540 3.9659840 5.7224485 0.241 0.809739

Bcell 3.2848382 26.7046619 2.0568351 1.597 0.110258

Mono 1.8988906 6.6784815 2.3243187 0.817 0.413948

Gran 0.2364654 1.2667637 1.4086527 0.168 0.866688

coef exp(coef) se(coef) z Pr(>|z|)

DNAmAgeHannum 0.0220819 1.0223275 0.0108290 2.039 0.04144 *

Age 0.0090386 1.0090796 0.1077026 0.084 0.93312

CD8.naive -0.0006915 0.9993088 0.0002189 -3.160 0.00158 **

CD8T 1.4718636 4.3573479 1.3139208 1.120 0.26263

CD4T 2.2060835 9.0800847 1.6830342 1.311 0.18993

NK 1.2504273 3.4918347 5.7290047 0.218 0.82722

Bcell 3.3766516 29.2725898 2.0750274 1.627 0.10368

Mono 1.6244790 5.0757741 2.3072241 0.704 0.48138

Gran 0.2427433 1.2747413 1.4129094 0.172 0.86359

**Detailed results for LBC1936**


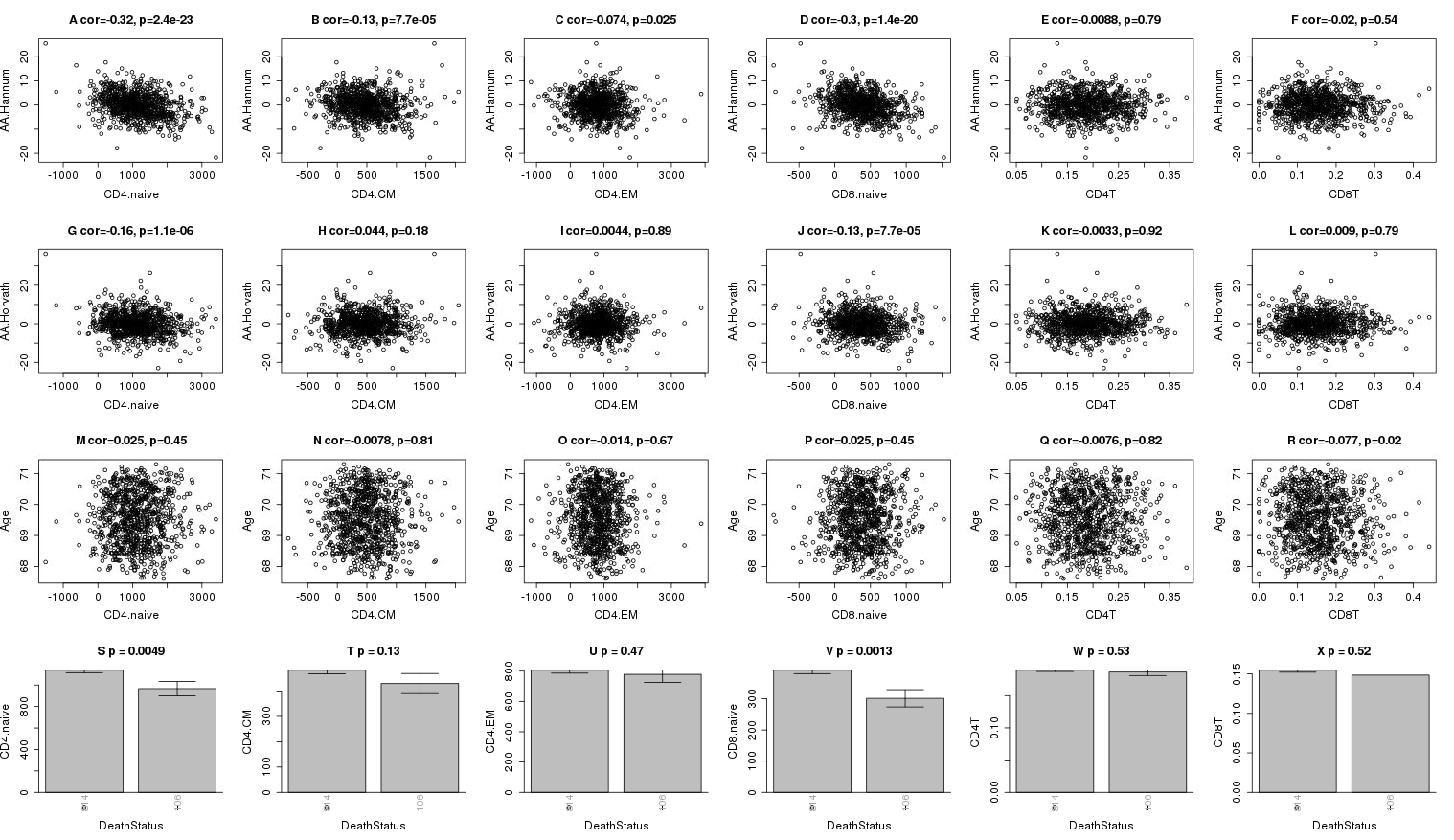


**LBC1936: T cell abundance measures versus age acceleration, chronological age and death status.**

A-F (first row): Age acceleration according to the Hannum predictor (y-axis) versus estimated abundance measures of naive CD4+ T cells (A), central memory CD4+T cells (B), effector memory CD4+T cells (C), naive CD8+ T cells (D), proportion of CD4+ T cells (E), and proportion of CD8+ T cells (F). G-L (second row) presents analogous results for the age predictor by Horvath. M-R (third row) analogous results for chronological age (y-axis). S-X (fourth row) relationship between death status (x-axis) and the various cell abundance measures. Each panel in the last row reports a non-parametric group comparison test (Kruskal Wallis test p-value). Each bar plot shows the mean value and 1 standard error.

Multivariate Cox regression analysis

coef exp(coef) se(coef) z Pr(>|z|)

DNAmAgeHorvath 3.070e-02 1.031e+00 1.532e-02 2.004 0.0451 *

Age 1.104e-02 1.011e+00 1.221e-01 0.090 0.9279

CD8.naive -7.829e-04 9.992e-01 3.110e-04 -2.517 0.0118 *

CD8T -1.708e+00 1.813e-01 2.140e+00 -0.798 0.4248

CD4T -2.187e+00 1.123e-01 2.755e+00 -0.794 0.4273

NK 5.283e+00 1.969e+02 7.153e+00 0.739 0.4602

Bcell 2.521e+00 1.244e+01 2.918e+00 0.864 0.3876

Mono -4.868e-01 6.146e-01 4.101e+00 -0.119 0.9055

Gran -3.797e-01 6.841e-01 2.461e+00 -0.154 0.8774

coef exp(coef) se(coef) z Pr(>|z|)

DNAmAgeHannum 3.530e-02 1.036e+00 1.936e-02 1.823 0.0683 .

Age 9.058e-03 1.009e+00 1.220e-01 0.074 0.9408

CD8.naive -6.942e-04 9.993e-01 3.271e-04 -2.122 0.0338 *

CD8T -1.535e+00 2.155e-01 2.133e+00 -0.720 0.4718

CD4T -2.129e+00 1.189e-01 2.766e+00 -0.770 0.4414

NK 6.326e+00 5.587e+02 7.274e+00 0.870 0.3845

Bcell 2.741e+00 1.550e+01 2.916e+00 0.940 0.3472

Mono -7.262e-01 4.837e-01 4.083e+00 -0.178 0.8588

Gran -3.074e-01 7.353e-01 2.460e+00 -0.125 0.9005

**Detailed results for NAS**


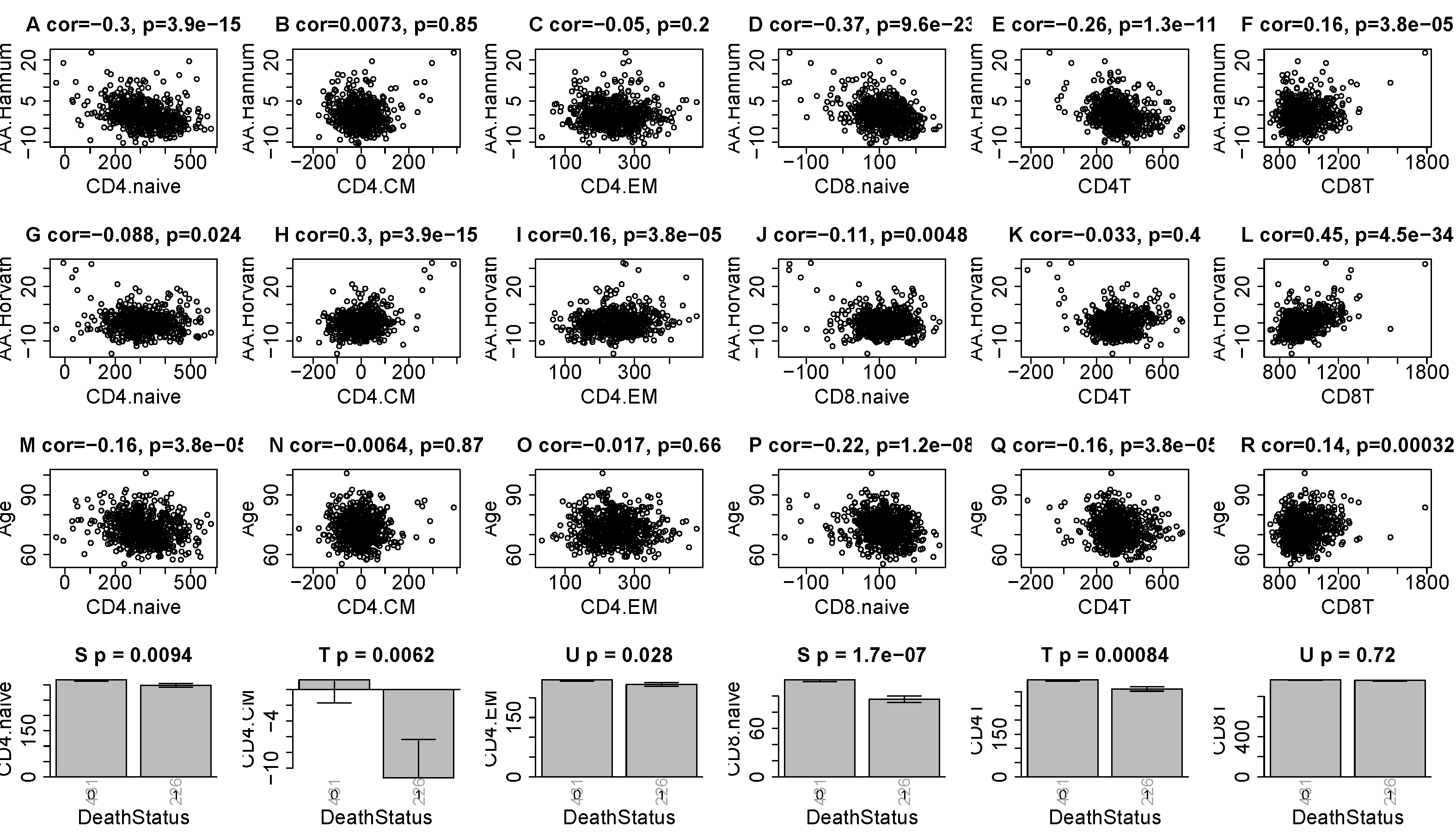


**NAS: T cell abundance measures versus age acceleration, chronological age and death status.**

A-F (first row): Age acceleration according to the Hannum predictor (y-axis) versus estimated abundance measures of naive CD4+ T cells (A), central memory CD4+T cells (B), effector memory CD4+T cells (C), naive CD8+ T cells (D), proportion of CD4+ T cells (E), and proportion of CD8+ T cells (F). G-L (second row) presents analogous results for the age predictor by Horvath. M-R (third row) analogous results for chronological age (y-axis). S-X (fourth row) relationship between death status (x-axis) and the various cell abundance measures. Each panel in the last row reports a non-parametric group comparison test (Kruskal Wallis test p-value). Each bar plot shows the mean value and 1 standard error.

**Multivariate Cox model for all cause mortality in the NAS**

coef exp(coef) se(coef) z Pr(>|z|)

DNAmHorvath 0.017668 1.017825 0.012802 1.380 0.167549

Age -0.049968 0.951260 0.022034 -2.268 0.023341 *

CD8.naive -0.006621 0.993401 0.001728 -3.830 0.000128 ***

CD8T -0.206863 0.813131 2.775660 -0.075 0.940591

CD4T 4.043814 57.043488 2.090590 1.934 0.053077 .

NK -1.400341 0.246513 2.384057 -0.587 0.556950

Bcell -0.355003 0.701171 2.675543 -0.133 0.894443

Mono 0.361896 1.436049 3.142699 0.115 0.908323

Gran 2.605220 13.534208 2.090938 1.246 0.212780

coef exp(coef) se(coef) z Pr(>|z|)

DNAmHannum 0.024200 1.024495 0.015744 1.537 0.124270

Age -0.054291 0.947157 0.022895 -2.371 0.017724 *

CD8.naive -0.006124 0.993895 0.001704 -3.594 0.000326 ***

CD8T -1.011573 0.363647 2.841725 -0.356 0.721862

CD4T 3.118775 22.618653 2.048449 1.523 0.127882

NK -2.362739 0.094162 2.413361 -0.979 0.327568

Bcell -1.284943 0.276666 2.818637 -0.456 0.648481

Mono -0.697360 0.497898 3.176068 -0.220 0.826208

Gran 1.533983 4.636606 2.148977 0.714 0.475339

**Detailed results for FHS**


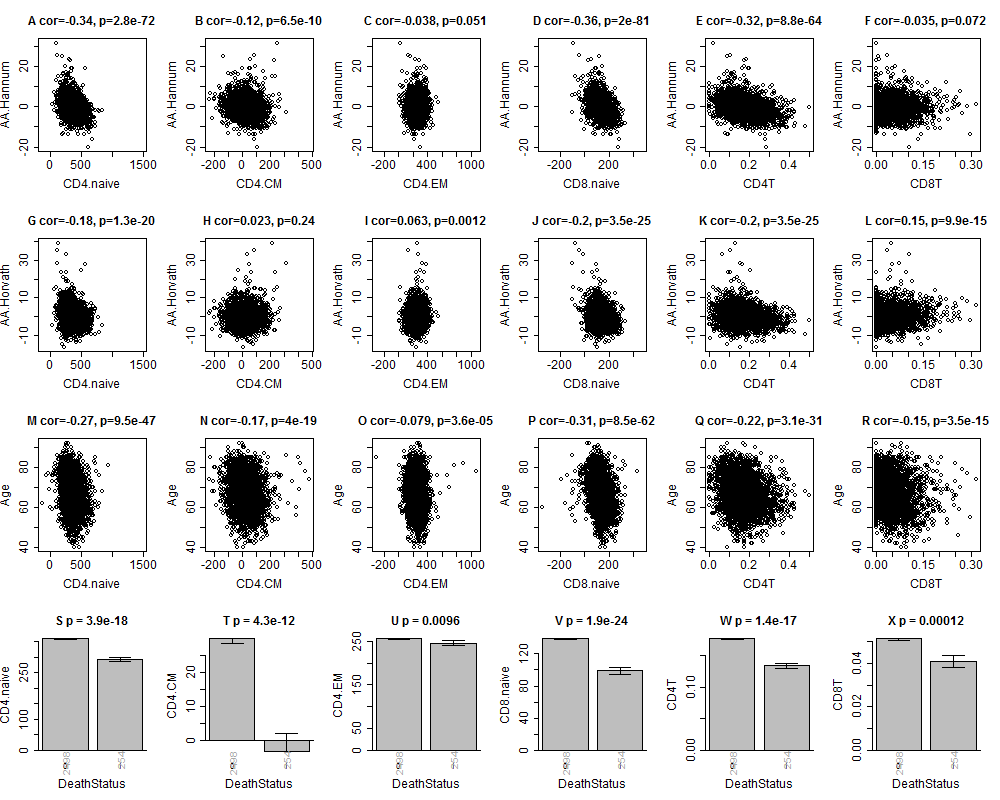


**FHS: T cell abundance measures versus age acceleration, chronological age and death status.**

A-F (first row): Age acceleration according to the Hannum predictor (y-axis) versus estimated abundance measures of naive CD4+ T cells (A), central memory CD4+T cells (B), effector memory CD4+T cells (C), naive CD8+ T cells (D), proportion of CD4+ T cells (E), and proportion of CD8+ T cells (F). G-L (second row) presents analogous results for the age predictor by Horvath. M-R (third row) analogous results for chronological age (y-axis). S-X (fourth row) relationship between death status (x-axis) and the various cell abundance measures. Each panel in the last row reports a non-parametric group comparison test (Kruskal Wallis test p-value). Each bar plot shows the mean value and 1 standard error.

Multivariate Cox regression models

coef exp(coef) se(coef) robust se z Pr(>|z|)

DNAmAgeHorvath 2.816e-02 1.029e+00 1.247e-02 1.330e-02 2.117 0.03424 *

Age -1.074e-01 8.982e-01 3.817e-02 3.530e-02 -3.043 0.00234 **

CD8.naive -4.670e-03 9.953e-01 1.683e-03 1.666e-03 -2.804 0.00505 **

CD8T 5.855e+00 3.491e+02 3.573e+00 3.497e+00 1.674 0.09405 .

CD4T 5.381e+00 2.172e+02 3.242e+00 3.338e+00 1.612 0.10693

NK 3.186e+00 2.419e+01 3.370e+00 3.452e+00 0.923 0.35606

Bcell 5.751e+00 3.145e+02 3.607e+00 3.896e+00 1.476 0.13987

Mono 1.111e+01 6.655e+04 3.756e+00 4.235e+00 2.623 0.00873 **

Gran 9.017e+00 8.240e+03 3.143e+00 3.138e+00 2.873 0.00407 **

Lab 1.123e-01 1.119e+00 1.605e-01 1.552e-01 0.724 0.46909

coef exp(coef) se(coef) robust se z Pr(>|z|)

DNAmAgeHannum 4.394e-02 1.045e+00 1.399e-02 1.462e-02 3.006 0.002648 **

Age -1.181e-01 8.886e-01 3.843e-02 3.548e-02 -3.329 0.000873 ***

CD8.naive -4.418e-03 9.956e-01 1.684e-03 1.630e-03 -2.711 0.006713 **

CD8T 4.441e+00 8.483e+01 3.636e+00 3.582e+00 1.240 0.215121

CD4T 3.950e+00 5.195e+01 3.287e+00 3.444e+00 1.147 0.251361

NK 1.611e+00 5.007e+00 3.456e+00 3.634e+00 0.443 0.657523

Bcell 3.859e+00 4.740e+01 3.689e+00 4.021e+00 0.960 0.337293

Mono 8.879e+00 7.177e+03 3.831e+00 4.289e+00 2.070 0.038460 *

Gran 7.018e+00 1.116e+03 3.218e+00 3.269e+00 2.147 0.031797 *

Lab 9.731e-02 1.102e+00 1.598e-01 1.515e-01 0.642 0.520741
