# Supplementary material for: DNA methylation age of blood predicts all-cause mortality in later life
Source: Genome Biol. 2015 Jan 30;16(1):25. doi: 10.1186/s13059-015-0584-6 (PMC4350614; doi:10.1186/s13059-015-0584-6)
Supplement: Additional file 4: — Contains a table of the associations of Δ age (per 5 years) with mortality in LBC1921 and LBC1936 after individual adjustment for covariates. The basic adjustment model controls for age and sex. A separate Cox model adjusting for age, sex, and a single covariate was analyzed along with a saturated model that included age, sex, and all covariates together. [file 13059_2015_584_MOESM4_ESM.docx]

Additional data file 4: Associations of Δ_age_ (per 5 years) with mortality in LBC1921 and LBC1936 after individual adjustment for covariates. The basic adjustment model controls for age and sex. A separate Cox model adjusting for age, sex, and a single covariate was analysed along with a saturated model that included age, sex, and all covariates together.

|  | **LBC1921** | | | **LBC1936** | | |
| --- | --- | --- | --- | --- | --- | --- |
|  | **HR** | **95% CI** | **P** | **HR** | **95% CI** | **P** |
| **Hannum** |  |  |  |  |  |  |
| Basic adjustment | 1.17 | [1.06, 1.30] | 2.7x10^-3^ | 1.22 | [1.01, 1.48] | 0.04 |
| Smoking (ex/current vs. never) | 1.15 | [1.04, 1.27] | 8.5x10^-3^ | 1.21 | [1.00, 1.45] | 0.04 |
| Education (years) | 1.17 | [1.05, 1.30] | 3.3x10^-3^ | 1.20 | [0.99, 1.46] | 0.06 |
| Age-11 IQ (per SD) | 1.14 | [1.02, 1.27] | 0.02 | 1.16 | [0.95, 1.43] | 0.14 |
| Occupational social class (per category) | 1.16 | [1.05, 1.29] | 4.5x10^-3^ | 1.16 | [0.96, 1.42] | 0.13 |
| *APOE* (e4 vs. no e4 allele) | 1.17 | [1.05, 1.30] | 3.6x10^-3^ | 1.24 | [1.03, 1.50] | 0.03 |
| Cardiovascular disease (yes vs. no) | 1.16 | [1.05, 1.29] | 4.4x10^-3^ | 1.23 | [1.01, 1.48] | 0.04 |
| High Blood Pressure (yes vs. no) | 1.17 | [1.05, 1.30] | 3.5x10^-3^ | 1.22 | [1.01, 1.47] | 0.04 |
| Diabetes (yes vs. no) | 1.17 | [1.05, 1.30] | 3.7x10^-3^ | 1.22 | [1.01, 1.48] | 0.04 |
| Fully adjusted model | 1.08 | [0.96, 1.21] | 0.18 | 1.12 | [0.91, 1.38] | 0.30 |
|  |  |  |  |  |  |  |
| **Horvath** |  |  |  |  |  |  |
| Basic adjustment | 1.16 | [1.05, 1.28] | 2.6x10^-3^ | 1.19 | [1.02, 1.38] | 0.03 |
| Smoking (ex/current vs. never) | 1.15 | [1.04, 1.27] | 5.9x10^-3^ | 1.17 | [1.01, 1.36] | 0.04 |
| Education (years) | 1.16 | [1.05, 1.28] | 3.5x10^-3^ | 1.18 | [1.01, 1.37] | 0.04 |
| Age-11 IQ (per SD) | 1.14 | [1.03, 1.26] | 0.01 | 1.19 | [1.01, 1.40] | 0.03 |
| Occupational social class (per category) | 1.15 | [1.05, 1.27] | 4.6x10^-3^ | 1.18 | [1.01, 1.38] | 0.04 |
| *APOE* (e4 vs. no e4 allele) | 1.17 | [1.06, 1.29] | 2.1x10^-3^ | 1.20 | [1.02, 1.40] | 0.02 |
| Cardiovascular disease (yes vs. no) | 1.15 | [1.04, 1.27] | 6.5x10^-3^ | 1.19 | [1.02, 1.39] | 0.03 |
| High Blood Pressure (yes vs. no) | 1.16 | [1.05, 1.28] | 3.7x10^-3^ | 1.18 | [1.02, 1.38] | 0.03 |
| Diabetes (yes vs. no) | 1.16 | [1.05, 1.28] | 2.9x10^-3^ | 1.18 | [1.02, 1.37] | 0.03 |
| Fully adjusted model | 1.10 | [0.99, 1.22] | 0.09 | 1.17 | [0.98, 1.38] | 0.08 |

LBC: Lothian Birth Cohort, SD: standard deviation, CI: Confidence Interval
