# Supplementary material for: DNA methylation age of blood predicts all-cause mortality in later life
Source: Genome Biol. 2015 Jan 30;16(1):25. doi: 10.1186/s13059-015-0584-6 (PMC4350614; doi:10.1186/s13059-015-0584-6)
Supplement: Additional file 5: — Contains a figure with the meta-analysis results of sex-stratified, age-adjusted models of Δ age against mortality. [file 13059_2015_584_MOESM5_ESM.docx]

Additional data file 5: Meta-analysis results of sex-stratified, age-adjusted models of Δ_age_ against mortality.^*^


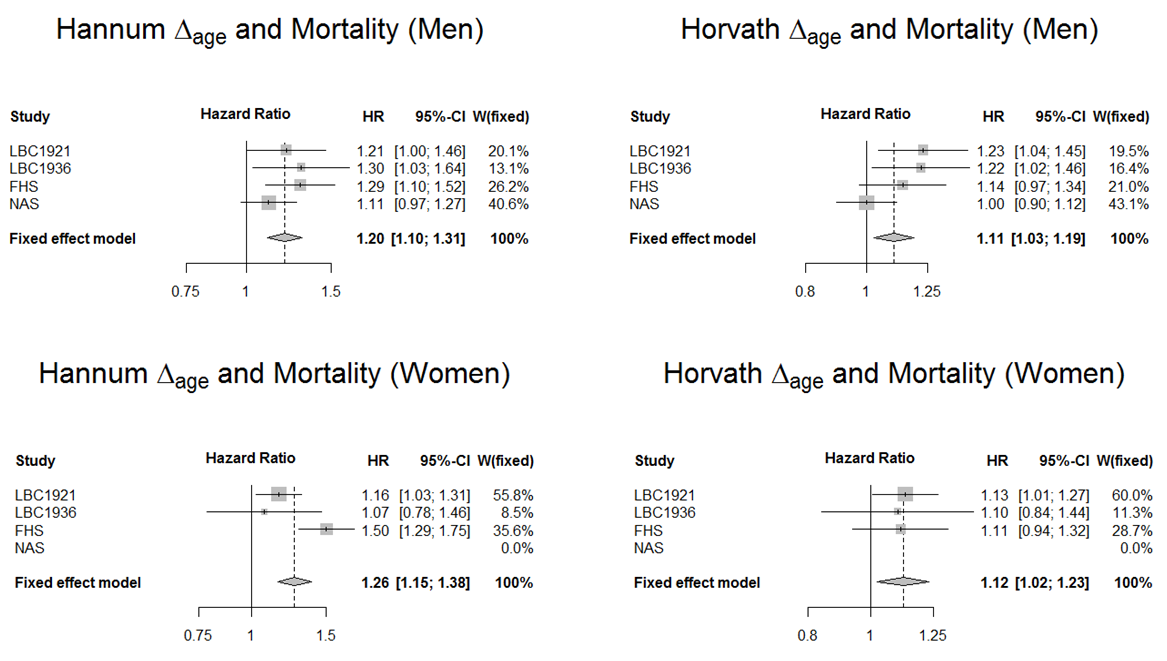


LBC: Lothian Birth Cohort, NAS: Normative Aging Study, FHS: Framingham Heart Study. NAS had only male participants.

^*^Analysis of FHS data were also adjusted for laboratory batch and family structure.
