# Supplementary material for: DNA methylation age of blood predicts all-cause mortality in later life
Source: Genome Biol. 2015 Jan 30;16(1):25. doi: 10.1186/s13059-015-0584-6 (PMC4350614; doi:10.1186/s13059-015-0584-6)
Supplement: Additional file 7: — Contains a table with the associations of Δ age with known mortality risk factors. Separate linear regression analyses were performed for each covariate. All models adjusted for sex except for NAS, which only had male participants. Analysis of FHS data was adjusted for laboratory batch and family structure. [file 13059_2015_584_MOESM7_ESM.docx]

Additional data file 7: Association of Δ_age_ with known mortality risk factors. Separate linear regression analyses were performed for each covariate. All models adjusted for sex except for NAS, which only had male participants. Analysis of FHS data were adjusted for laboratory batch and family structure.

|  | **LBC1921** | | | **LBC1936** | | | **FHS** | | | **NAS** | | |
| --- | --- | --- | --- | --- | --- | --- | --- | --- | --- | --- | --- | --- |
|  | **Beta^*^** | **SE** | **P** | **Beta^*^** | **SE** | **P** | **Beta^*^** | **SE** | **P** | **Beta^*^** | **SE** | **P** |
| **Hannum Δ_age_ (per 5 years)** |  |  |  |  |  |  |  |  |  |  |  |  |
| Sex (female) | -0.63 | 0.10 | 4.1x10^-9^ | -0.52 | 0.06 | 1.9x10^-15^ | -0.48 | 0.07 | 6.9x10^-50^ | - | - | - |
| Smoking (current/ex vs. never) | 0.19 | 0.10 | 0.06 | 0.01 | 0.07 | 0.85 | 0.05 | 0.04 | 0.22 | 0.06 | 0.08 | 0.49 |
| Education (years) | -0.01 | 0.02 | 0.53 | -0.07 | 0.03 | 0.01 | - | - | - | 0.01 | 0.01 | 0.55 |
| Age-11 IQ (per SD) | -0.03 | 0.06 | 0.64 | -0.09 | 0.03 | 0.01 | - | - | - | - | - | - |
| Occupational social class (per category) | 0.01 | 0.06 | 0.85 | 0.11 | 0.04 | 2.9x10^-3^ | - | - | - | - | - | - |
| *APOE* (e4 vs. no e4 allele) | 0.16 | 0.12 | 0.16 | -0.11 | 0.07 | 0.15 | - | - | - | -0.03 | 0.09 | 0.72 |
| Cardiovascular disease (yes vs. no) | 0.11 | 0.11 | 0.35 | -0.05 | 0.08 | 0.48 | -0.13 | 0.06 | 0.02 | -0.01 | 0.08 | 0.92 |
| High Blood Pressure (yes vs. no) | 0.11 | 0.10 | 0.31 | 0.13 | 0.07 | 0.06 | -0.03 | 0.04 | 0.41 | -0.04 | 0.08 | 0.61 |
| Diabetes (yes vs. no) | 0.39 | 0.24 | 0.10 | -0.06 | 0.12 | 0.62 | 0.12 | 0.06 | 0.03 | 0.10 | 0.10 | 0.32 |
|  |  |  |  |  |  |  |  |  |  |  |  |  |
| **Horvath Δ_age_ (per 5 years)** |  |  |  |  |  |  |  |  |  |  |  |  |
| Sex (female) | -0.42 | 0.12 | 4.6x10^-4^ | -0.20 | 0.08 | 0.01 | -0.35 | 0.04 | 7.3x10^-16^ | - | - | - |
| Smoking (current/ex vs. never) | 0.22 | 0.12 | 0.06 | 0.04 | 0.08 | 0.62 | 0.07 | 0.04 | 0.11 | -0.10 | 0.10 | 0.30 |
| Education (years) | -0.02 | 0.02 | 0.51 | -0.06 | 0.03 | 0.06 | - | - | - | 0.01 | 0.02 | 0.59 |
| Age-11 IQ (per SD) | -0.05 | 0.06 | 0.45 | 0.01 | 0.04 | 0.79 | - | - | - | - | - | - |
| Occupational social class (per category) | 0.003 | 0.07 | 0.96 | 0.04 | 0.04 | 0.30 | - | - | - | - | - | - |
| *APOE* (e4 vs. no e4 allele) | -0.07 | 0.13 | 0.60 | -0.13 | 0.08 | 0.13 | - | - | - | 0.12 | 0.11 | 0.28 |
| Cardiovascular disease (yes vs. no) | 0.19 | 0.13 | 0.14 | -0.05 | 0.09 | 0.61 | -0.27 | 0.06 | 6.9x10^-6^ | -0.05 | 0.10 | 0.61 |
| High Blood Pressure (yes vs. no) | 0.12 | 0.12 | 0.32 | 0.14 | 0.08 | 0.06 | -0.22 | 0.04 | 8.6 x10^-7^ | 0.05 | 0.10 | 0.62 |
| Diabetes (yes vs. no) | 0.19 | 0.28 | 0.49 | 0.11 | 0.14 | 0.43 | 0.04 | 0.06 | 0.48 | 0.23 | 0.12 | 0.05 |

LBC: Lothian Birth Cohort, NAS: Normative Aging Study, FHS: Framingham Heart Study, SD: standard deviation. * Unstandardised betas.
