# Supplementary material for: DNA methylation age of blood predicts all-cause mortality in later life
Source: Genome Biol. 2015 Jan 30;16(1):25. doi: 10.1186/s13059-015-0584-6 (PMC4350614; doi:10.1186/s13059-015-0584-6)

Additional data file 8: Model fitting in BSGS to create residuals from a regression of predicted age against chronological age. A quadratic chronological age term was included in the adolescent model along with chronological age and sex (red line). A linear model adjusting for age and sex was included in the adult model (blue line). BSGS: Brisbane Systems Genetics Study.
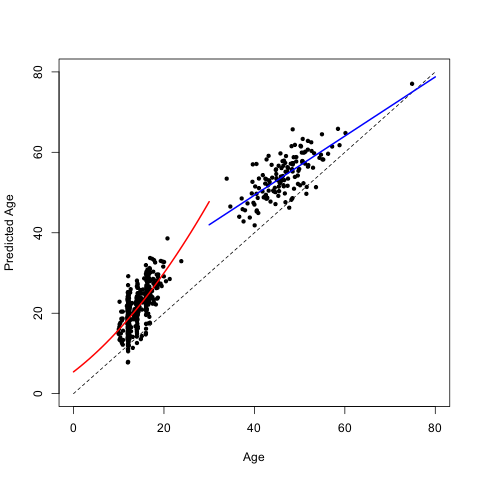

Supplement: Additional file 8: — Contains a figure illustrating the model fitting in BSGS to create residuals from a regression of predicted age against chronological age. A quadratic chronological age term was included in the adolescent model along with chronological age and sex (red line). A linear model adjusting for age and sex was included in the adult model (blue line). [file 13059_2015_584_MOESM8_ESM.docx]
